# Nasolacrimal obstruction concomitant to cataract: diagnosis and management in the preoperative period of cataract surgery

**DOI:** 10.5935/0004-2749.20210044

**Published:** 2021

**Authors:** Luis Henrique Zucoloto, Marina Trentin Regueiro Artioli, Denise Moreira Zornoff, Alicia Galindo-Ferreiro, Silvana Artioli Schellini

**Affiliations:** 1 Faculdade de Medicina, Universidade Estadual Paulista “Júlio de Mesquita Filho”, Botucatu, SP, Brazil; 2 Department of Ophthalmology, Otorhinolaryngology and Head and Neck Surgery, Faculdade de Medicina, Universidade Estadual Paulista “Júlio de Mesquita Filho”, Botucatu, SP, Brazil; 3 Computer Technical Service and Distance Education Center, Faculdade de Medicina, Universidade Estadual Paulista “Júlio de Mesquita Filho”, Botucatu, SP, Brazil; 4 Department of Ophthalmology, Rio Hortega University Hospital, Dulzaina St, Valladolid, Spain

**Keywords:** Cataract, Cataract extraction, Endophthalmitis, Lacrimal duct obstruction, Dacryocystitis, Internet-based intervention, Surveys and questionnaires, Catarata, Extração de catarata, Endoftalmite, Obstrução dos ductos lacrimais, Dacriocistite, Intervenção baseada em internet, Inquéritos e questionários

## Abstract

**Purpose:**

Concomitant nasolacrimal duct obstruction can occur in cataract carriers, which increases the risk of postoperative endophthalmitis. The primary aim of this study is to evaluate the knowledge of Brazilian cataract surgeons on the diagnosis and management of cataracts associated with nasolacrimal duct obstruction.

**Methods:**

This survey was based on a questionnaire involving Brazilian cataract surgeons that was conducted from March to April 2018. Data were collected on the participant’s profile, time and experience in ophthalmic practice, previous training in diagnosis and management of nasolacrimal duct obstruction, and background with endophthalmitis after cataract surgery in patients with nasolacrimal duct obstruction. All data were entered into an Excel spreadsheet and analyzed according to the frequency of occurrence.**Results:** Ninety-one ophthalmologists answered the questionnaire. Most (63.7%) had been performing cataract surgery for >10 years, and most (84.6%) received training to diagnose and handle nasolacrimal duct obstruction during their medical residence training. Nasolacrimal duct obstruction was investigated in the preoperative period of the cataract by lacrimal sac expression test (53.8%) or by irrigation of the tear pathways (23.1%). Nasolacrimal duct obstruction was treated with antibiotic eye drops by 47.2% of respondents. Seventy-eight percent of surgeons indicate usually performing lacrimal surgery prior to the intraocular surgery, waiting for 4 to 6 weeks to proceed with the cataract surgery. The procedure of choice for treating nasolacrimal duct obstruction prior to cataract surgery was dacryocystorhinostomy (88.4%). Most participants recognized the need for a protocol to assist in the detection and management of nasolacrimal duct obstruction in cataract carriers.

**Conclusion:**

Improvement in the diagnosis and management of nasolacrimal duct obstruction concomitant to cataract is needed, as this is a risk factor for endophthalmitis.

## Abstract (pt)

**Objetivo:**

Portadores de catarata podem apresentar concomitantemente obstrução do ducto lacrimo-nasal (DLN), com risco de desenvolver endoftalmite no pós-operatório da facectomia. O objetivo do presente estudo é apresentar as percepções dos cirurgiões de catarata sobre a propedêutica e a conduta frente a pacientes com obstrução do ducto lacrimo-nasal concomitante com catarata.

**Métodos:**

Trata-se de uma pesquisa baseada em um questionário envolvendo cirurgiões brasileiros de catarata, realizado no período de março a abril de 2018. Foram levantados dados sobre o perfil dos participantes, o tempo e a experiencia da prática oftalmológica, o treinamento prévio para diagnóstico e tratamento da obstrução do ducto lacrimo-nasal e os conhecimentos de endoftalmite após cirurgia de catarata. Todos os dados foram inseridos em planilha Excel e analisados de acordo com a frequência de ocorrência.

**Resultados:**

Noventa e um oftalmologistas responderam ao questionário. A maioria (63,7%) deles realiza cirurgias de catarata há mais de 10 anos e a maioria (84,6%) recebeu treinamento para diagnóstico e tratamento da obstrução do ducto lacrimo-nasal durante o curso de residência médica. A pesquisa da obstrução crônica do ducto lacrimo-nasal no pré-operatório da catarata é feita pelo teste do refluxo de secreção pelos pontos lacrimais (53,8%) ou por irrigação das vias lacrimais (23,1%). A obstrução do ducto lacrimo-nasal é tratada com colírios antibióticos por 47,2% dos respondentes. Para os portadores de obstrução do ducto lacrimo-nasal , 78% indicam a desobstrução das vias lacrimais previamente à facectoma, aguardando de 4 a 6 semanas para tal. O procedimento de escolha para tratar a obstrução do ducto lacrimo-nasal antes da facectomia é a dacriocistorrinostomia (88,4%). A necessidade de um protocolo para auxiliar na detecção e tratamento da obstrução do ducto lacrimo-nasal em portadores de catarata é reconhecida pela maioria dos participantes deste estudo. **Conclusão: É** necessário melhorar a propedêutica e o manejo da catarata em portador de obstrução do ducto lacrimo-nasal porque esse é um fator de risco para endoftalmite.

## INTRODUCTION

Cataracts occur in 37.5% of patients with nasolacrimal duct obstruction (NLDO)^(1)^. In these cases, bacterial stasis in the lacrimal sac secondary to chronic NLDO can lead to the development of postoperative endophthalmitis after cataract surgery^(2)^. NLDO has been detected by irrigation and macrodacryocystography in patients who developed endophthalmitis, confirming that lacrimal obstruction is a significant risk factor for endophthalmitis after cataract surgery^(3)^. Therefore, for patients who are undergoing lens extraction, routine propaedeutic examination of the tear outflow system for NLDO screening is recommended.

Despite the clear possibility of concurrent NLDO and cataract and the influence of this association as a risk factor for endophthalmitis, several cases remain unrecognized prior to cataract extraction^(3,4)^.This study was conducted with the purpose of evaluating the standard protocols followed by Brazilian cataract surgeons in terms of the diagnosis and handling of NLDO prior to cataract extraction.

## METHODS

The research protocol used in the present study was approved by the Research Ethics Committee of the Botucatu Medical School-UNESP, and a consent form was obtained from all participants.

This was a qualitative research study based on an online questionnaire administered to 2050 cataract surgeons who were affiliated members of the Brazilian Association of Refractive Surgery Cataract, from March to April 2018. The doctors were invited to participate in the survey by e-mail. To improve participation, another e-mail was sent after 2 weeks. The anonymity of the participants was guaranteed by removing the data from the internet protocol address and adding an automatic numeric value allocated to each computer connected to the web at the time of the response analysis. The questionnaire was prepared by the authors and can be accessed at http://www3.fmb.unesp.br/questionarios/index.php/713437/lang-pt-BR.

The questionnaire was performed using the adaptively configured open-source software Lyme Survey and consisted of 20 multiple-choice questions, with the possibility of additional questions or comments depending on the respondent’s answers. Participant were asked to provide information on demographic distribution, place of work (private or public service), length of practice and experience with cataract surgery, number of cataract surgeries performed per month, oculoplastic training rec eived during education, routine propaedeutic examination and management of patients with NLDO concurrent with cataract, previous experience and number of postcataract surgeries performed in patients with endophthalmitis associated with NLDO, and outcome of endophthalmitis cases due to concomitant NLDO and cataract. All principal survey questions were configured with mandatory answers.

Responses were entered into an Excel spreadsheet (Microsoft Corp., Redmond, WA, USA) and analyzed according to the frequency of occurrence.

## RESULTS

Ninety-one Brazilian ophthalmologists answered the questionnaire, with a geographic distribution as follows: 49 (53.8%) in the southeast, 20 (22%) in the northeast, 14 (15.4%) in the south, 5 (5.49%) in the midwest, 1 (1.1%) in the north, and 2 (2%) unreported place of residence. Forty-two (46.1%) respondents were working in the private medical system, 2 (2.2%) in only the public system, and 47 (51.6%) in both systems.

Fifty-eight (63.7%) respondents had been performing cataract surgery for >10 years, 21 (23.1%) for >5 to <10 years, and 12 (13.2%) for <5 years. Regarding the number of cataract surgeries per year, 40 (44%) participants reported usually performing >200 surgeries, 34 (37.3%) >50 to <200 surgeries, and 17 (18.7%) <50 surgeries per year.

Seventy-seven (84.6%) respondents had received training in lacrimal surgery during their medical residency course, 12 (13.2%) in a specialization course, 11 (12.1%) during individualized study, 9 (9.9%) during fellowship, and 5 (5.5%) during their undergraduate degree program. With regard to lacrimal propaedeutic examinations, despite previous training, 42 (46.1%) reported moderate experience, 36 (39.5%) little experience, 11 (12.1%) reported having good experience, and 2 (2.2%) reported no experience in this field.

Forty-nine (53.8%) respondents reported usually treating only simple outflow lacrimal drainage affection and referring complicated cases, 29 (31.9%) referred all cases to oculoplastic doctors, 12 (13.2%) treated all cases, and 1 (1.1%) had no cases.

According to 72 (79.1%) respondents, fewer than 5% of patients had cataract and NLDO, 8 (8.8%) respondents reported that about 30% of their patients had concomitant affection, and 11 (12, 1%) said they did not have this information.

To detect possible NLDO in the preoperative period of cataract, 49 (53.8%) respondents reported usually investigating the obstruction using lacrimal sac expression and observing secretion reflux through the lacrimal puncta; 21 (23.1%) evaluated the lacrimal system using irrigation and lacrimal sac expression; 11 (12.1%) performed irrigation only if the patient complains of tearing or discharge; 2 (2.2%) used irrigation in all cases; 2 (2.2%) performed dacryocystography; and 6 (6.6%) believed that verifying the patency of the NLDO preoperatively is not necessary.

Seventy-one (78%) participants believed that with NLDO, the lacrimal obstruction should be removed prior to cataract surgery or other intraocular surgeries; 10 (11%) agreed with this approach only in some cases, such as with active obstruction revealed by abundant discharge and epiphora and confirmed by dacryocystography; 7 (7.7%) did not know if lacrimal surgery should be performed before cataract removal; and 3 (3.3%) responded that this procedure is not necessary.

In the cases diagnosed as NLDO, 56 (61.5%) respondents stated that they always indicate or perform outflow lacrimal procedure before cataract surgery, 19 (20.9%) do not indicate or perform, and 13 (14.3 %) indicate or perform only when there are frequent infections or abundant reflux of secretion, when dacryoliths are present, and in those who do not respond to dilatation and lavage of the lacrimal pathways.

When there is no proven NLDO concurrent with cataract, the preferred approach is dacryocystorhinostomy (61 respondents; 88.4%), followed by dacryocystectomy (5 respondents; 7.2%). Three participants (3.3%) leave the surgical indication to the oculoplastic specialist, and 1 (1.1%) believes the obstruction can be resolved with a massage. When performing dacryocystorhinostomy, 38 (62.3%) participants wait 6 weeks before evaluating the normalization of the conjunctival flora to proceed with the cataract surgery and 23 (37.7%) wait 4 weeks between the two procedures. Of those who prefer dacryocystectomy, 3 (60%) wait 4 weeks between surgeries and 2 (40%) wait 6 weeks.

With regard to the preference for using antibiotic eye drops as clinical treatment for NLDO, 43 (47.2%) always use this option, 34 (37.4%) do not use this treatment, and 11 (12.1%) use antibiotic eye drops in patients who are unable to undergo dacryocystorhinostomy or who have an active infection or spontaneous purulent discharge and require urgent cataract surgery.Four (4.4%) participants reported attending at least one patient who developed endophthalmitis after cataract extraction and had concomitant NLDO. Eleven (12.1%) never had any case, but they knew of colleagues who had such cases; the rest (76-83.5%) had no experience with this type of situation. Of the 15 (16.5%) participants who encountered patients with endophthalmitis after cataract surgery with concomitant NLDO, 7 (46.7%) reported that endophthalmitis occurred earlier than 1 month after cataract surgery and 1 (6, 7%) answered that endophthalmitis occurred later than 1 month after surgery. Seven (46.7%) did not have access to this information.

With regard to the final visual acuity of patients after endophthalmitis, 3 (20%) participants reported the visual acuity of cases remained better than 20/50, 4 (26.7%) reported that visual acuity was lower than 20/200, and 1 (6.7%) reported that the case continued to have no light perception. Eight (53.3%) participants did not have access to this information.

Finally, 69 (75.8%) participants reported that they believe it is important to have a preoperative protocol for the detection and management of cases of NLDO to reduce complications and the risk of poor outcome.

## DISCUSSION

The questionnaire administered to Brazilian cataract surgeons about the presence of NLDO concomitant to cataract revealed that, in general, the lacrimal system is not examined preoperatively, despite the recognized risk and severity of endophthalmitis.

The profiles of the 91 respondents indicated that about half belonged to the southeast region of Brazil, which is consistent with the greater distribution of doctors in this region. Most respondents had >10 years of experience performing cataract surgery (63.7%), and about half had as much experience in cataract treatment, performing >200 cataract surgeries per year.

Most surgeons believe that the lacrimal system should be unblocked before cataract surgery. However, only those surgeons who performed fewer than 200 cataract surgeries per year effectively indicate/perform the lacrimal surgery before the intraocular surgery.The vast majority of respondents had received lacrimal training, especially during their medical residency. However, likely because of the low incidence of NLDO reported in the general population (3%-6.6%)^(5)^ participants recognize that they have little experience with this type of disease, in both diagnosis and management. Half of the respondents treat only simple cases and refer difficult cases to other specialists, and 29.7% of them refer all cases.

We found that the frequency of cataract carriers and concomitant NLDO is quite low^(6)^, as participants reported that this affected <5% of their patients.Most (53.8%) Brazilian cataract surgeons reported that their preferred approach for screening of possible NLDO is lacrimal sac expression upon examination of secretion reflux. An Indian study reported that this test is routinely used for screening of lacrimal obstruction, with a sensitivity of 93.2% and specificity of 99.3%^(7)^. In our opinion, and also the opinion of Indian surgeons, irrigation of the lacrimal system for screening is a time-consuming and costly test^(7)^.Almost 80% of participants recognize that unblocking the lacrimal system is required before cataract surgery or other intraocular surgery, which is in accordance with other reports^(3)^. However, many participants were not aware of the problem and did not investigate NLDO before cataract surgery. In India, nearly 8% of the respondents felt that it is not necessary to check for NLDO preoperatively^(8)^, which is, of course, not the ideal practice.Dacryocystorhinostomy or dacryocystectomy can be used to clear the lacrimal system. Brazilian cataract surgeons reported that dacryocystectomy is a much less preferred option, but this technique is an excellent option, particularly for older patients, as tear production declines with age.

After lacrimal surgery, the anterior segment of the microbial flora should be normalized to avoid the risk of endophthalmitis secondary to NLDO. However, there is no defined time frame for this procedure. The average time for normalization varies according to the type and amount of bacteria and whether a stent was placed after dacryocystorhinostomy; this time period ranges from 3.9 to 7 weeks^(9)^. Thus, 62% of Brazilian doctors consider 6 weeks as an adequate interval between lacrimal surgery and cataract surgery. In another region, only 19% of surgeons reported waiting for 6 weeks to proceed with the cataract surgery^(8)^.Clinical NLDO management using antibiotic eye drops before cataract surgery was the choice of almost half of the participants (47.2%). A Japanese study demonstrated temporary control of conjunctival bacterial flora using antibiotic eye drops during the 3 weeks before cataract surgery^(9)^. However, if the lacrimal system is not opened definitively, flora control is not permanent, and this option may be considered only in urgent cases, such as in glaucoma secondary to cataract or in patients with poor surgical conditions.

Only 4.4% of participants reported assisting a patient with concomitant cataract and NLDO who developed endophthalmitis after cataract surgery. Despite the low incidence of endophthalmitis after cataract surgery (about 0.18%), half of endophthalmitis cases occurring after cataract surgery present with concomitant NLDO^(3,10)^. Endophthalmitis occurred <1 month after cataract surgery, corroborating the findings of other researchers, who reported the onset of endophthalmitis symptoms in >90% of cases as <30 days^(10)^, despite the possibility of having later cases.

Although only a few participants provided information about the final visual acuity of patients with endophthalmitis, the outcome of poor visual acuity is already known in patients with endophthalmitis, a finding that was also repeated in cases with concomitant NLDO^(10)^. Finally, the need for a preoperative protocol for screening and management of NLDO concomitant with cataract was recognized by most of the participants.

Preoperative cataract evaluation should involve the investigation of NLDO using the test of lacrimal sac expression. If NLDO is detected, dacryocystorhinostomy or dacryocystectomy should be performed before cataract surgery to prevent endophthalmitis. Placing greater emphasis on this subject in specialist training programs as well as ensuring better interaction between cataract surgeons and oculoplasts can reduce the occurrence of endophthalmitis in these cases.
